# Identification of cellular double-stranded RNAs in mammalian embryonic stem cells

**DOI:** 10.1261/rna.081048.126

**Published:** 2026-11

**Authors:** Katharina J. Kases, Pilar G. Marchante, Jeroen Witteveldt, Guillermo Peris, Grzegorz Kudla, Sara R. Heras, Sara Macias

**Affiliations:** 1 Institute of Immunology and Infection Research, Ashworth Laboratories, School of Biological Sciences, University of Edinburgh, Edinburgh EH9 3FL, United Kingdom; 2 GENYO, Centre for Genomics and Oncological Research: Pfizer/University of Granada/Andalusian Regional Government, PTS Granada, Granada 18016, Spain; 3 Department of Biochemistry and Molecular Biology II, Faculty of Pharmacy, University of Granada, Campus Universitario de Cartuja, Granada 18071, Spain; 4 Department of Computer Languages and Systems, Universitat Jaume I, Castellon de la Plana 12071, Spain; 5 MRC Human Genetics Unit, Institute for Genetics and Cancer, University of Edinburgh, Edinburgh EH4 2XU, United Kingdom

**Keywords:** dsRNA, ESC, TE, LINE, editing, antisense

## Abstract

Antiviral defense mechanisms are typically activated upon sensing virus-derived nucleic acids. During replication, viruses generate double-stranded RNA (dsRNA) intermediates that the innate immune system can sense, triggering several defense pathways. Conversely, mammalian cells avoid accumulating their own endogenous dsRNA to prevent activating these defense mechanisms. However, we demonstrate that mammalian embryonic stem cells (ESCs) accumulate endogenous dsRNA without activating these responses, as they lack all classical dsRNA-mediated antiviral pathways. To identify these endogenous dsRNAs, we have developed a two-step purification method that includes an antibody-based immunoprecipitation followed by RNase I treatment to remove single-stranded RNA regions that do not contribute to dsRNA formation. RNase I treatment results in an enrichment of overlapping sense/antisense transcripts containing A-to-I editing sites, suggesting successful purification of cellular dsRNA. Our refined protocol reveals that transposable elements (TEs), including evolutionary young elements from the LINE and LTR classes, are the predominant source of dsRNA in ESCs. This approach will be useful for investigating the role of dsRNA in disease settings, such as autoimmunity or cancer, where endogenous dsRNA accumulation has also been observed.

## INTRODUCTION

The accumulation of virus-derived double stranded RNA (dsRNA) in the cytoplasm of mammalian cells triggers multiple defense pathways. Viral dsRNA is sensed by the retinoic acid-inducible gene I (RIG-I)-like receptor (RLR) family of proteins, which activates the major antiviral innate immune pathway, the type I interferon (IFN) response. Within the RLR family, RIG-I recognizes short dsRNA containing a 5′-triphosphate group, while MDA5 recognizes longer dsRNA (Rehwinkel and Gack 2020). Upon dsRNA recognition, both these cytosolic sensors activate the transcription factor IRF3 driving the expression of type I interferons (IFNs) (Bourdon et al. 2025). Within the endosomal compartment, the Toll-like receptor 3 (TLR3) sensor also recognizes dsRNA leading to IFN expression through IRF3 activation (Chattopadhyay and Sen 2014). In addition to IFNs, viral dsRNA can induce other antiviral responses. For instance, upon binding to dsRNA, the protein kinase R (PKR) phosphorylates the translation initiation factor eIF2α, causing a general inhibition of cap-dependent mRNA translation (Dauber and Wolff 2009). DsRNA also activates the OAS/RNase L pathway resulting in a global degradation of both cellular and viral RNA (Lee et al. 2026). Although these dsRNA-activated responses are critical in providing antiviral defense, they can also be deleterious if inappropriately activated (Marchal et al. 2014; Crow and Casanova 2024).

Considering that the innate immune system cannot discriminate virus from host-derived dsRNA molecules, mammalian cells avoid accumulating endogenous dsRNA to prevent aberrant immune activation. Mammalian cells possess several mechanisms to prevent dsRNA accumulation, one of the most studied being based on RNA modifications. The post-transcriptional editing of adenosine to inosines (A-to-I) within dsRNA by ADAR1 disrupts base-pairing, preventing their sensing by MDA5 or PKR (Rehwinkel and Mehdipour 2025). Most editing sites are contained in the noncoding regions of RNAs, overlapping with repetitive sequences derived from transposable elements (TEs), predominantly SINE/Alu elements (Porath et al. 2017). *N*^6^-methyladenosine (m^6^A) modifications have also been implicated in suppressing the RLR-dependent IFN response, as loss of the m^6^A writer METTL3 resulted in an increased IFN signature, suggesting that m^6^A modifications prevent dsRNA formation (Gao et al. 2020). Similarly, incorporation of pseudouridines into mRNA prevents sensing by PKR (Anderson et al. 2010). Additional mechanisms include the activity of RNA helicases, such as DHX9, which has been proposed to inhibit the formation of TE-derived dsRNA in complex with other dsRNA binding proteins, including ADAR1 or DGCR8 (Aktaş et al. 2017; Murayama et al. 2024; Gázquez-Gutiérrez et al. 2026). Finally, Staufen-mediated mRNA decay degrades mRNAs that contain dsRNA structures in their 3′UTRs, formed by intra- or intermolecular base-pairing (Park and Maquat 2013). More recently, a distinct splicing pathway, termed SOS splicing, was shown to remove inverted terminal repeat elements adopting dsRNA hairpin structures, thereby restoring the function of genes disrupted by TEs (Zhao et al. 2026).

The repetitive nature of TEs, along with the fact that they can be transcribed in sense or antisense orientation as part of the genes where they are inserted, makes them prime candidates for dsRNA formation. As such, TEs have emerged as a major source of dsRNA in different disease settings, including cancer, senescence and autoimmune diseases (Gázquez-Gutiérrez et al. 2021). Transcription initiated from intrinsic TE promoters is epigenetically silenced across most healthy somatic tissues to minimize the risk of de novo transposition and consequent genomic instability. A notable exception occurs during early development, when specific TE families are derepressed and can be transcribed as full-length copies from their own promoters. Such is the importance of TE expression in embryogenesis, that they orchestrate the transcriptional profile during early development (DiRusso and Clark 2023; Oomen et al. 2025).

To identify mammalian cellular dsRNA, multiple methods have been developed. DsRNA can be immunoprecipitated using anti-dsRNA antibodies from either purified RNA or total cell extracts (Dhir et al. 2018; Wiatrek et al. 2019; Gao et al. 2020; Ghosh et al. 2020; Krieger et al. 2024; Witteveldt et al. 2025; Gázquez-Gutiérrez et al. 2026). One of the best-studied antibodies is the monoclonal J2, which is highly specific against dsRNA, and shows no cross-reactivity against dsDNA, RNA/DNA hybrids, ssRNA, or ssDNA. J2 was initially reported to recognize dsRNA species with a minimum length of 40 base pairs (bp); however, more sensitive recent studies indicate that it can bind dsRNA helices as short as 14 bp, with the binding affinity increasing with dsRNA length (Schonborn et al. 1991; Bou-Nader et al. 2025). Besides antibodies, dsRNA-binding proteins, such as dsRNA sensors, can also be used to identify cellular dsRNA. For instance, in vitro RNase protection assays with MDA5 and immunoprecipitation of MDA5 have been used to identify cellular dsRNA that can bind this sensor (Ahmad et al. 2018; Clapes et al. 2021). Similarly, PKR immunoprecipitation following formaldehyde cross-linking has been successfully used to identify cellular dsRNA (Kim et al. 2018). Nonetheless, using sensors for dsRNA identification has limitations, as they only capture the subset of transcripts preferentially bound by a given sensor, rather than the entire population of dsRNA in the cell.

Here, we show that although mouse ESCs accumulate significantly higher levels of dsRNA than somatic cells, they fail to respond to these as they lack all major antiviral responses to dsRNA. These include the type I interferon (IFN) response as well as functional PKR and OAS/RNase L pathways. To identify the endogenous ESC-dsRNA population, we have developed a two-step purification method using the anti-dsRNA antibody J2 followed by RNase I digestion. This approach was inspired by previous reports where purified total RNA was treated with RNase I prior J2 immunoprecipitation to identify virus-derived dsRNA (Decker et al. 2019; Price et al. 2022). Instead, we purify cellular dsRNA from extracts using the J2 antibody, followed by RNase I treatment to increase the proportional dsRNA yield. Our strategy exploits the differential sensitivity of dsRNA to the structure-specific RNA nucleases. Whereas dsRNA is largely resistant to single-strand-specific nucleases such as RNase I, it remains sensitive to cleavage by the dsRNA-specific endoribonuclease RNase III. Our results demonstrate that the two-step purification improves the detection of cellular dsRNA by enriching for both sense, antisense and edited transcripts, while removing non-dsRNA sequences. We further show that dsRNA in ESCs derive from TEs and are enriched for the youngest LINE and LTR subfamilies. These evolutionary young elements are typically longer and have accumulated fewer mutations, features that increase their propensity to form highly complementary and therefore more stable dsRNA. This approach aims to improve both the sensitivity and specificity of dsRNA identification across diverse biological contexts, including homeostasis, infection, and pathological states associated with aberrant dsRNA accumulation, such as autoimmunity and cancer.

## RESULTS

### mESCs lack dsRNA-mediate immune responses

We and others have previously shown that mouse ESCs cannot trigger the IFN response upon dsRNA stimulation (Wang et al. 2013; Witteveldt et al. 2019, 2025). Yet, the status of other major dsRNA-induced innate immune pathways in mouse ESCs remains poorly defined. To address this, we transfected mESCs with exogenous dsRNA [poly(I:C)] and compared their response to their differentiated derivatives. As expected, ESC differentiation resulted in decreased expression of the pluripotency markers *Nanog* and *Oct4* (*Pou5f1*) and increased expression of the differentiation markers *Acta2* and *Tagln*, as shown by RT-qPCR. None of these markers were significantly affected by the dsRNA challenge (Fig. 1A). In differentiated cells, dsRNA stimulation activated the expression of IFNs (*Ifnb1*) and interferon-stimulated genes (ISGs, *Oas1* and *Stat1*), while no increase in expression was observed in ESCs, as expected (Fig. 1B). In agreement with these findings, differentiated cells exhibited phosphorylated IRF3 upon dsRNA stimulation, indicating successful RLR-mediated signaling (Fig. 1C; Supplemental Fig. 1). Next, we assessed whether ESCs preserved other dsRNA-mediated antiviral responses, specifically the PKR and OAS/RNase L pathways. We monitored the phosphorylation of the kinase PKR by western blot, as this kinase auto-phosphorylates upon dsRNA binding. Interestingly, PKR phosphorylation was detected only in differentiated cells, with no changes observed in ESCs upon dsRNA transfection (Fig. 1D; Supplemental Fig. 1). Finally, dsRNA stimulation also activated the OAS/RNase L system in differentiated cells, leading to degradation of ribosomal RNA (rRNA). In contrast, ESCs did not respond to dsRNA, maintaining the integrity of both 28S and 18S rRNA (Fig. 1E).

**FIGURE 1.**
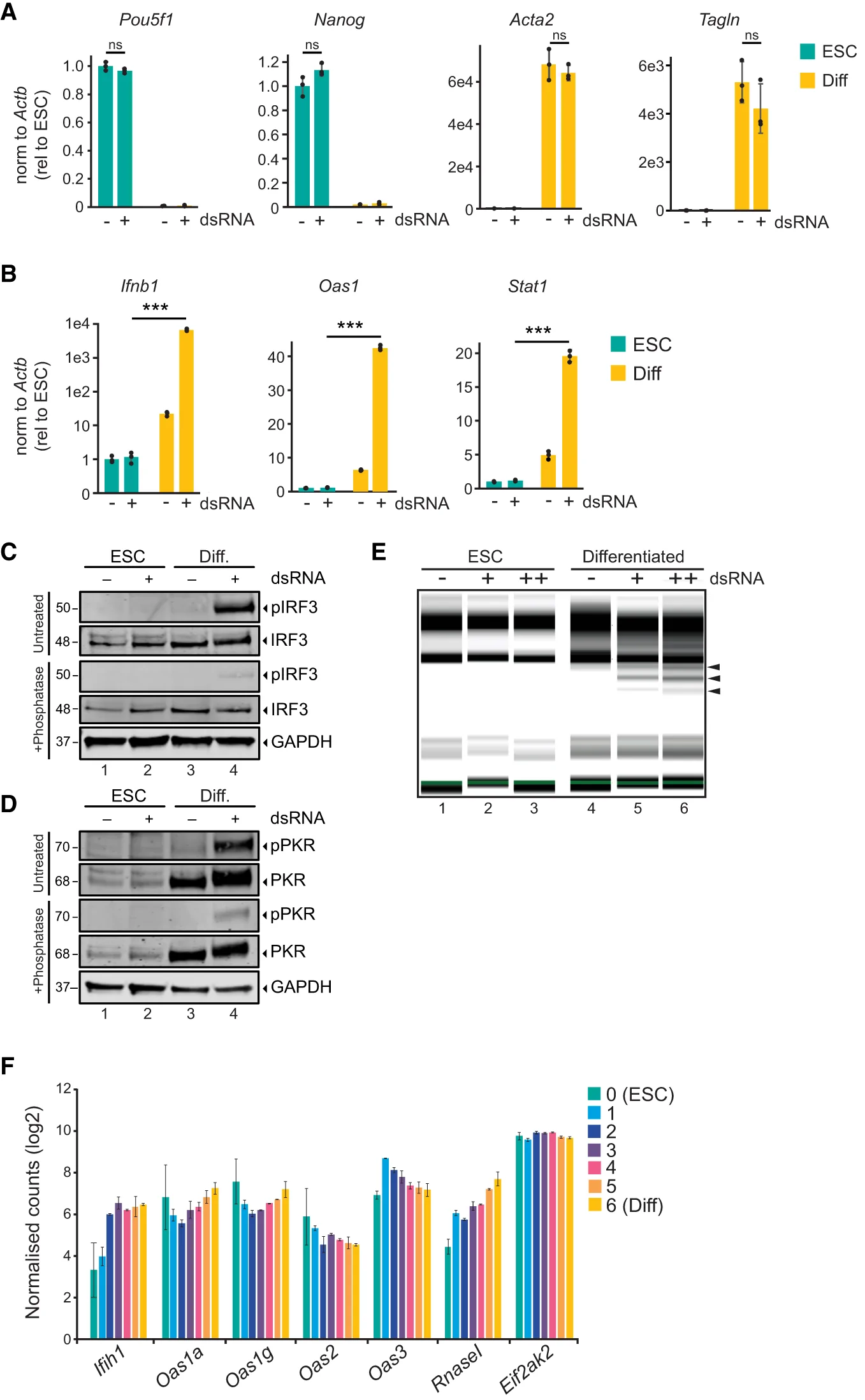
Characterization of dsRNA-mediated antiviral response in ESCs and differentiated cells. (*A*) Expression of the pluripotency markers *Pou5f1* and *Nanog* and differentiation markers *Acta2* and *Tagln* in ESCs and differentiated cells was measured by RT-qPCR, comparing mock and dsRNA-stimulated cells. *Actb* was used as a normalizer. Data represent the average of three biological replicates ±SD relative to untreated ESC. One-way ANOVA was used to calculate significant differences between dsRNA-treated samples within each cell line, followed by a Tukey HSD. (*) *P* ≤ 0.05, (**) *P* ≤ 0.01, (***) *P* ≤ 0.001. (*B*) Expression of *Ifnb1* and interferon stimulated genes *Oas1* and *Stat1* in ESCs and differentiated cells upon dsRNA or mock stimulation by RT-qPCR analysis. *Actb* was used as a normalizer. Data represent the average of three biological replicates ± SD relative to untreated ESC. One-way ANOVA was used to calculate significant differences between dsRNA-treated cell lines, followed by a Tukey HSD. (*) *P* ≤ 0.05, (**) *P* ≤ 0.01, (***) *P* ≤ 0.001. (*C*) Western blot analysis of phosphorylated IRF3 levels in ESCs and differentiated cells on control- and dephosphorylated western blots. GAPDH serves as a loading control. (*D*) Western blot analysis of phosphorylated PKR in ESCs and differentiated cells on control- and dephosphorylated western. Tubulin serves as a loading control. (*E*) rRNA electrophoresis from ESCs and differentiated cells, mock and dsRNA-stimulated. (+) represents lower dsRNA and (++) higher dsRNA stimulation. (*F*) mRNA expression levels of RNA-sensors during differentiation to ectoderm. Expression is average of three biological replicates ± SD.

We also measured the expression of known dsRNA sensors in ESCs and differentiated cells. For this, we analyzed a transcriptomic data set of ESCs differentiating to ectoderm (Fig. 1F; Sladitschek and Neveu 2019). Both *Ifih1* (MDA5) and *Rnasel* (RNase L) were poorly expressed in ESCs and their expression increased after differentiation (Fig. 1F). We confirmed these observations in our ESC model system and differentiated cells using high-throughput RNA-sequencing (Supplemental Fig. 1C). In contrast, *Eif2ak2* (PKR) was highly expressed across all the stages of pluripotency and differentiation (Fig. 1F; Supplemental Fig. 1C), which agrees with the observation of being the only sensor detectable in ESCs by western blot or proteomic analysis (Witteveldt et al. 2019). As the PKR pathway seems to also be inhibited in ESCs, an alternative mechanism may exist to control its activation. Interestingly, PACT has recently been identified as a negative regulator of PKR function and our data show that it is highly expressed in ESCs (Ahmad et al. 2025).

These findings indicate that all the major dsRNA-mediated antiviral responses are inactive in ESCs and are only acquired upon differentiation. These differences may be, in part, caused by the low expression of conventional dsRNA sensors in ESCs, or the presence of inhibitors that limit their function. This led us to conclude that mESCs represent a dsRNA-permissive environment.

### Accumulation and purification of dsRNA in ESCs

Compared with somatic cells, mESCs accumulate endogenous dsRNA. However, because ESCs lack the major antiviral pathways triggered by dsRNA, this accumulation does not trigger any unwanted immune activation. In contrast, these antiviral pathways become activated upon differentiation. This observation led us to hypothesize that endogenous dsRNA levels must be reduced during differentiation to prevent its aberrant recognition as foreign. To test this, we compared the levels of dsRNA in ESCs versus in vitro differentiated cells by flow cytometry using anti dsRNA antibodies (J2). Our findings confirmed that ESCs but not differentiated cells accumulate endogenous dsRNA (Fig. 2A,B). We verified the specificity of the dsRNA signal by treating cells with RNase III, a dsRNA-specific nuclease, before probing with J2. RNase III treatment resulted in a reduction of the signal, approaching the level found in differentiated cells (Fig. 2A,B; Supplemental Fig. 2). Immunofluorescence microscopy with the anti-dsRNA antibody confirmed that ESCs accumulate substantial amounts of dsRNA and that this accumulation is lost upon differentiation (Fig. 2C). These results demonstrate that endogenous dsRNA accumulate in ESCs but are lost upon differentiation, coincident with the acquisition of functional dsRNA-mediated responses.

**FIGURE 2.**
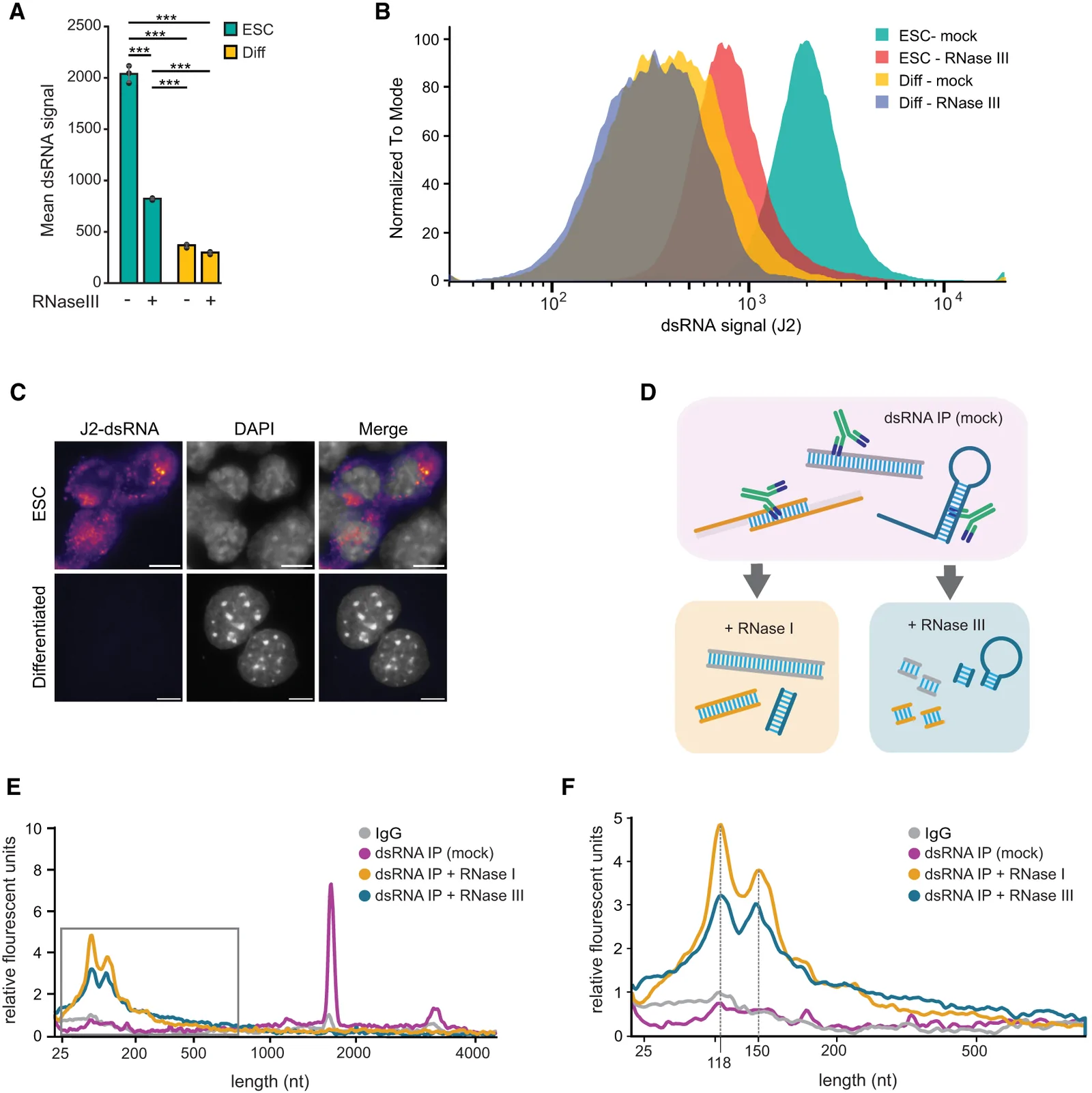
DsRNA quantification in ES and differentiated cells. (*A*) Flow cytometry analysis against dsRNA using the dsRNA-specific antibody J2 in ESC versus differentiated (diff) cells in the presence or absence of RNase III. RNase III treatment tests specificity of J2 antibody signal. Data represent the average of three biological replicates ± SD. One-way ANOVA was used to calculate significant differences among comparisons, followed by a Tukey HSD. (*) *P* ≤ 0.05, (**) *P* ≤ 0.01, (***) *P* ≤ 0.001. (*B*) Representative examples of normalized Flow cytometry intensity in ESCs and differentiated cells after mock and RNase III treatment. For all replicates and gating strategy, see Supplemental Figure 2A. (*C*) Immunofluorescence for dsRNA. J2 antibody was used to detect dsRNA, and DAPI to stain the nucleus. Scale bar, 10 µm. (*D*) Setup of dsRNA-immunoprecipitation using the J2 antibody. DsRNA bound to J2 were either mock treated, treated with RNase I or RNase III prior to purification and followed high-throughput RNA sequencing. (*E*) Bioanalyzer electropherogram of purified RNAs J2-IP using the different treatments shown in *D*. (*F*) Detailed view of Bioanalyzer electropherogram showing accumulation of shorter fragments upon treatment with RNase I and III.

To identify the ESC-enriched dsRNA, we developed a two-step purification method, consisting of dsRNA immunoprecipitation from cell extracts using the anti-dsRNA antibody J2, followed by RNase I digestion to remove single-stranded RNA (ssRNA) regions. RNase I is an endoribonuclease that specifically degrades ssRNA without sequence bias (Kennell 2002). In cells, dsRNA can arise by complementary interactions either *in cis* within a single transcript or *in trans* between distinct RNA molecules. Intramolecular base-pairing of complementary sequences can give rise to hairpin structures, whereas intermolecular pairing between sense and antisense transcripts can generate dsRNA duplexes. These structures may be perfectly base-paired or may contain mismatches resulting in bulges, internal loops and single-stranded overhangs (Fig. 2D, top). Given that the J2 antibody requires only a minimal double stranded stretch for binding, we hypothesized that besides this minimal duplex region, many of the immunoprecipitated RNAs would still retain substantial single stranded regions. To enrich for the dsRNA regions, we treated J2-immunoprecipitates with RNase I while still bound to the J2 antibody (Fig. 2D, bottom left). In parallel, to confirm that the immunoprecipitated RNAs represented dsRNA, we treated the J2-immunoprecipitated samples with RNase III, a dsRNA-specific endonuclease (Fig. 2D, bottom right). The RNA recovered after immunoprecipitation and treatment with RNase I, RNase III or no-treatment (“mock”) was analyzed on a Bioanalyzer alongside an IgG control. This confirmed the specificity of the J2 immunoprecipitation, as no RNA was recovered from the IgG control (Fig. 2E). Compared with the mock-treated dsRNA IP, both RNase treatments reduced the size of the predominant immunoprecipitated RNA species (Fig. 2F; Supplemental Fig. 3A). Overall, these RNA profiles demonstrated that the J2 immunoprecipitation was specific, and that treatment with either nuclease enriched for shorter RNA fragments.

### RNase I treatment improves dsRNA detection

Immunopurified dsRNA was subsequently identified using high-throughput RNA sequencing. Since the IgG negative control did not yield sufficient RNA for library preparation, we used the total RNA-seq data of the input samples to calculate the enrichment of immunoprecipitated dsRNA. Principal component analysis revealed that all the immunoprecipitated dsRNA samples clustered into distinct groups and, regardless of RNase treatment, were enriched for repetitive elements, including transposable elements (TEs), relative to the input (Supplemental Fig. 3B,C). Differential enrichment analyses showed an increase across all classes of transposable elements following dsRNA immunoprecipitation, namely DNA, SINE, LINE and LTR (Fig. 3A). However, TE classes exhibited different sensitivities to RNase treatments. For all classes, RNase III reduced the signal relative to the untreated (“mock”) dsRNA immunoprecipitations, confirming the presence of nucleic acids that are accessible and sensitive to dsRNA-specific RNases. However, unlike the other TE classes, LINEs remained similarly enriched upon RNase I treatment, suggesting that LINE-derived dsRNA species have the highest levels of complementarity, making them more resistant to RNase I degradation (Fig. 3A).

**FIGURE 3.**
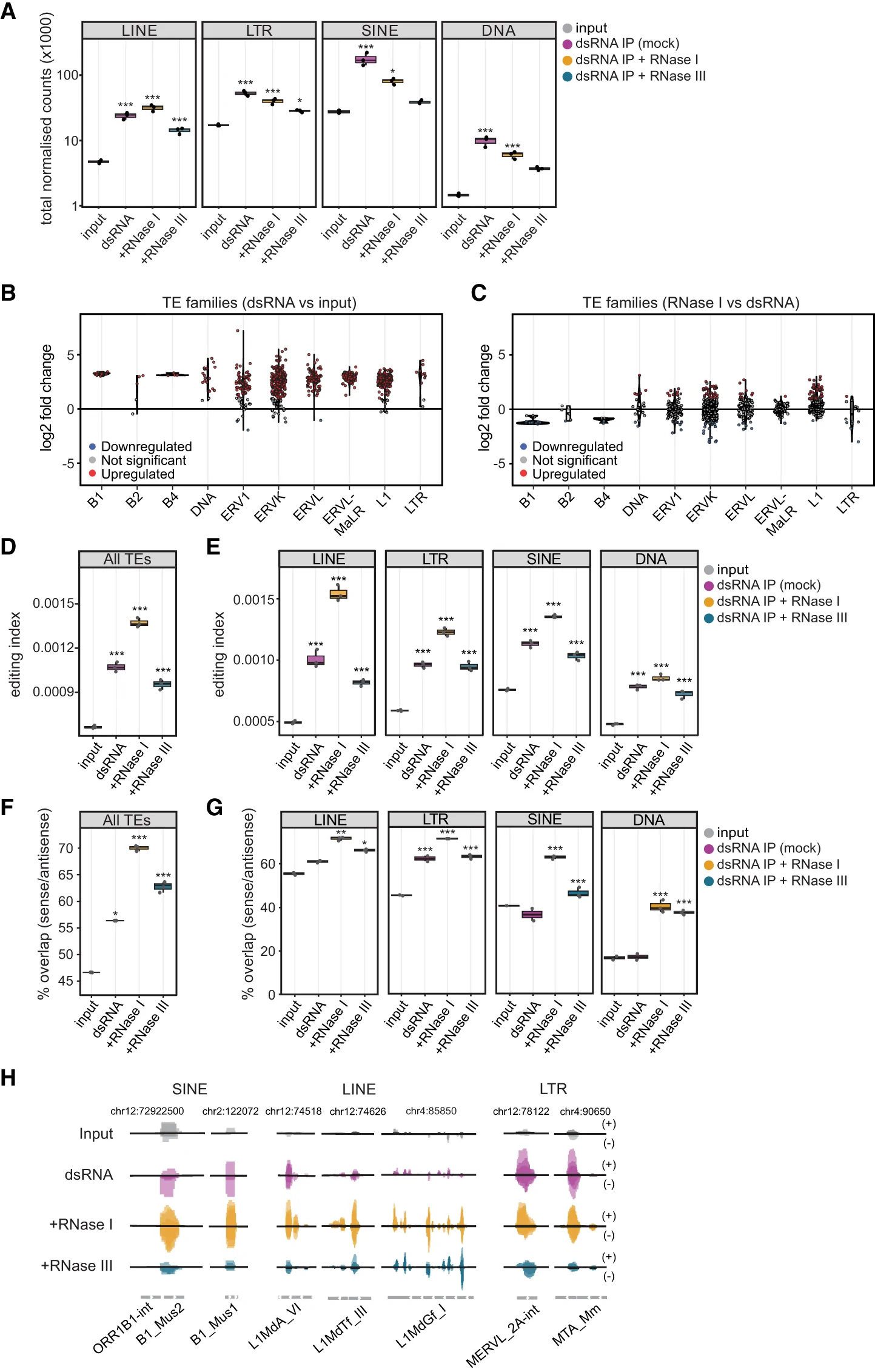
DsRNA are enriched in transposable elements with RNase treatment-specific differences. (*A*) Normalized counts of the four major classes of transposable elements compared to input controls. One-way ANOVA was used to calculate significant differences between input and immunoprecipitated samples, followed by a Tukey HSD. (*) *P* ≤ 0.05, (**) *P* ≤ 0.01, (***) *P* ≤ 0.001. (*B*) Violin plots showing differential enrichment (log_2_ fold change) of TE subfamilies within selected families for dsRNA IP (mock) versus input. Points represent individual subfamilies, colored by differential enrichment (red: upregulated, blue: downregulated, gray: not significant; log_2_FC > 1, adjusted *P* < 0.05). (*C*) Violin plots showing differential enrichment (log_2_ fold change) of TE subfamilies within selected families after RNase I treatment versus dsRNA (mock). Points represent individual subfamilies, colored by differential enrichment (red: upregulated, blue: downregulated, gray: not significant; log_2_FC > 1, adjusted *P* < 0.05). (*D*) Levels of A-to-I editing in retrotransposons for input and dsRNA-IPs. One-way ANOVA was used to calculate significant differences between input and immunoprecipitated samples, followed by a Tukey HSD. (*) *P* ≤ 0.05, (**) *P* ≤ 0.01, (***) *P*≤ 0.001. (*E*) Levels of A-to-I editing by TE class. Statistical analysis as in *D*. (*F*) Percentage of sense reads mapping to TEs with a corresponding antisense match, for input and dsRNA IPs. One-way ANOVA was used to calculate significant differences between input and immunoprecipitated samples, followed by a Tukey HSD. (*) *P* ≤ 0.05, (**) *P* ≤ 0.01, (***) *P* ≤ 0.001. (*G*) Sense/antisense overlap by TE class. Statistical analysis as in *F*. (*H*) Examples of specific TE locus. The combined normalized reads from the biological replicates mapped are shown, for inputs and dsRNA IPs. Above black line represents sense (+), below represents antisense reads (−). Chromosomal location of each locus is represented at the *top*.

DsRNA IPs displayed significant enrichment for most TE families compared to input (Fig. 3B). They were enriched in SINEs, including B1, B2, and B4 families; the LTR families ERV1, ERVK, ERVL, ERVL-MaLR, and LTR in addition to DNA and LINE families (Fig. 3B). In agreement with our previous analysis by TE class, RNase I treatment resulted in a reduction of the SINE families, B1, B2, and B4. A less consistent behavior was observed for LTR families, as some were enriched or lost upon RNase I treatment. In contrast, a more consistent behavior was observed with LINE subfamilies where a higher proportion remained enriched upon RNase I treatment. This suggests that LINEs form highly complementary dsRNA, while the others may be more frequently interrupted by ssRNA regions, making them more susceptible to RNase I cleavage (Fig. 3C). To further confirm that the TE families enriched after RNase I treatment were consistent with being dsRNA, we assessed their behavior in response to RNase III treatment. Most TE subfamilies showed significant depletion following dsRNA cleavage, supporting the conclusion that contain dsRNA species (Supplemental Fig. 3D).

To verify that the enriched sequences exhibit features consistent with dsRNA structures, we first quantified the adenosine-to-inosine (A-to-I) editing levels, as a marker of ADAR1 activity. The presence of editing would indicate that these sequences were present in cells as dsRNA, rather than being interactions formed in the extracts during cell lysis prior to purification. To this end, we measured the number of A-to-G nucleotide substitutions and compared this to other substitutions to ensure that these were true editing events rather than sequencing errors (Supplemental Fig. 4A). Next, we calculated the editing index, defined as the number of edited adenosines divided by the number of all possible editing sites. Across all transposons, the editing index was highest after RNase I treatment, supporting the observation that treatment enriches for dsRNA (Fig. 3D). All retrotransposon classes (LINE, LTR, and SINE) had a similar editing index after RNase I treatment, and it suggests that, in average, only 1 or 1.5 of every 1000 adenosines (0.1%–0.15%) were edited. The editing index was generally lower for DNA transposons (Fig. 3E).

As further evidence for the enrichment of dsRNA, we calculated the proportion of sense (+) reads that are overlapped by antisense (−) reads. Across all TE classes, immunoprecipitated dsRNA samples showed a significant enrichment of overlapping sequences, which further increased to 70% following RNase I treatment (Fig. 3F; Supplemental Fig. 4B). Among TE classes, LINEs and LTRs showed the highest proportions of overlapping reads across all treatments, while SINEs and DNA TEs showed the fewest sense/antisense overlap. The strongest overlap signal in LINE and LTR elements was observed following RNase I treatment (Fig. 3G). We observed a similar trend when analyzing normalized reads over individual TE copies. These confirmed an enrichment of complementary sequences in all dsRNA-immunoprecipitated samples, with the highest number of overlapping sense and antisense reads after RNase I treatment, and an overall lower signal after RNase III digestion (Fig. 3H). We hypothesized that the residual signal detected after RNase III treatment is partially due to protection of dsRNA by the J2 antibody or additional dsRNA-binding proteins. To test this, the purified RNA recovered after RNase III digestion was subjected to a second RNase III treatment. This second digestion resulted in smaller RNA fragment sizes, indicating that dsRNA bound by the J2 antibody, and possibly co-purified proteins, were partially protected from RNase III cleavage (Supplemental Fig. 4C). This aspect needs to be taken into consideration when interpreting the differential sensitivity of RNAs to these nucleases.

Taken together, these analyses indicate that the two-step dsRNA purification using RNase I digestion after J2-based immunoprecipitation enriches for dsRNA. This conclusion is supported by higher RNA-editing rates and a greater proportion of sense/antisense transcripts, although direct base-pairing interactions were not experimentally confirmed.

### Two-step purified dsRNA is enriched in young LINE-1 elements

To better understand what TE characteristics are associated with dsRNA formation and RNase I enrichment, we examined additional features of these elements. We first investigated the evolutionary age of the TEs enriched after RNase I treatment by determining the Kimura divergence of individual copies from the subfamily consensus sequence. This revealed that RNase I-enriched TEs had significantly lower Kimura divergence values, indicating that they were relatively younger elements (Fig. 4A). When analyzed by TE class, this effect was only significant for LINEs and LTRs, with a much more pronounced effect for LINEs. These findings suggest that younger LINE elements are more likely to form dsRNA and/or potentially generate more complementary, RNase I-resistant dsRNA structures (Fig. 4B).

**FIGURE 4.**
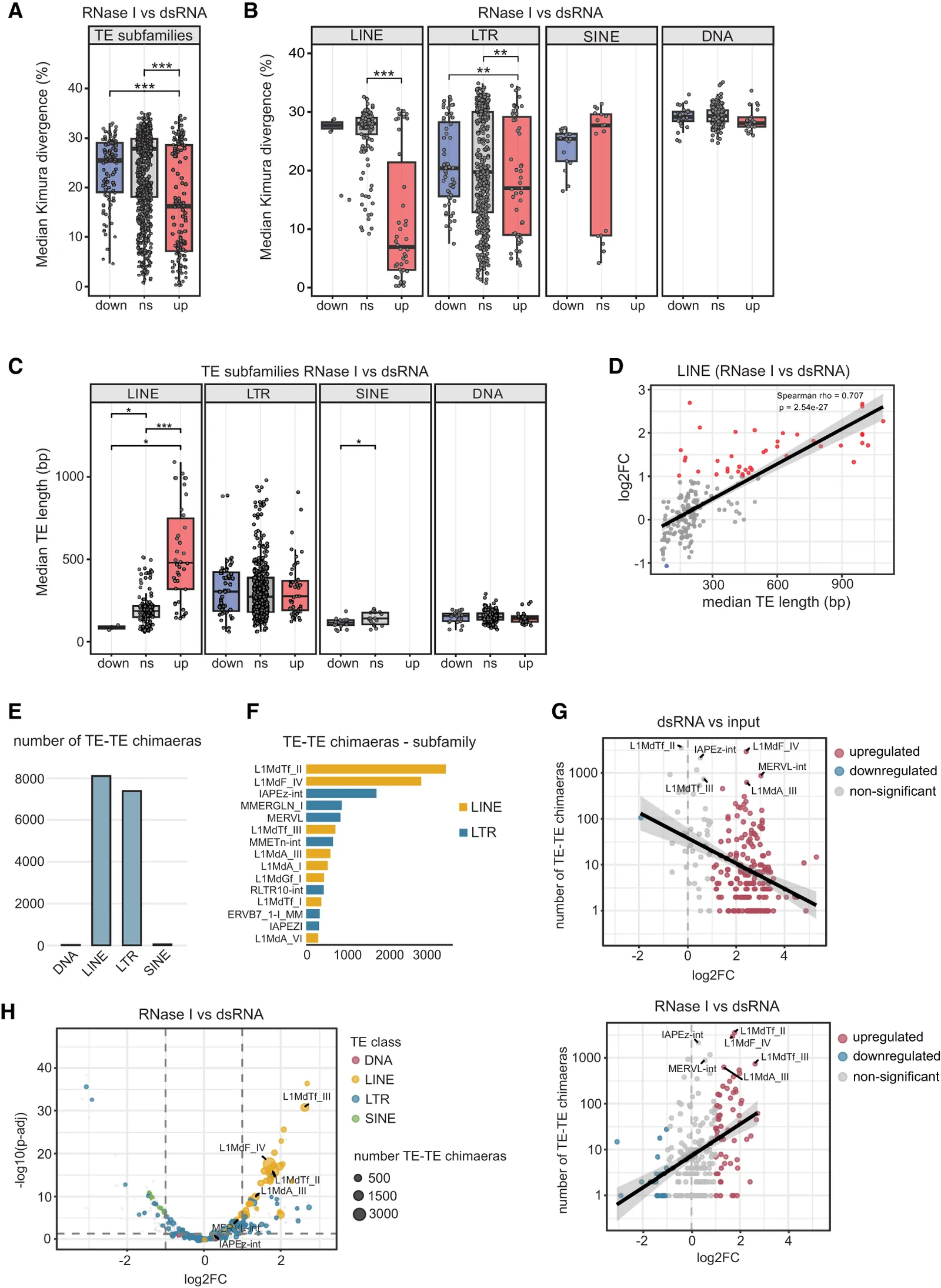
RNase I-enriched TEs are predominantly from young subfamilies. (*A*) Percentage of median Kimura divergence for enriched subfamilies after RNase I treatment versus mock or nontreated samples. (Up) Upregulated, (down) downregulated, (ns) nonsignificant. A Wilcoxon rank-sum test was used to test for significant differences between groups. (*B*) Percentage of median Kimura divergence by TE class. Statistics performed as in *A*. (*C*) Median TE length in base pairs (bp) by TE classes enriched upon RNase I treatment versus mock or nontreated. (*D*) Correlation analysis of LINE element-enrichment after RNase I treatment (log_2_FC, *Y*-axis) versus length (base pairs, bp; *x*-axis). Spearman correlation of ρ = 0.707 was significant (*P* < 0.05). (*E*) Number of TE-TE chimeras by TE class (DNA, LINE, LTR, SINE) in PARIS data sets in mouse ESCs. (*F*, *top*) TE subfamilies ranked by number of TE-TE chimeras in PARIS. Bars are colored by TE class: LINE (yellow) and LTR (blue). (*G*) Number of PARIS TE-TE chimeras (*y*-axis) plotted against dsRNA-IP log_2_FC (*x*-axis) for each TE subfamily across: dsRNA versus input (*top*), RNase I versus dsRNA (*bottom*). Significantly upregulated subfamilies are shown in red, downregulated in blue, and nonsignificant in gray. (*H*) Volcano plot of the RNase I versus dsRNA IP comparison, with point size proportional to the number of TE-TE chimeras in PARIS. Points are colored by TE class (DNA, red; LINE, yellow; LTR, blue; SINE, green).

In addition to the evolutionary age, we examined TE length and found that, on average, longer elements were more enriched after RNase I treatment. However, this pattern was specific to LINEs and not observed with the other TE classes (Fig. 4C). Consistently, LINE enrichment after ssRNA removal showed a significant positive correlation with element length (Fig. 4D). Collectively, these findings led us to conclude that in ESCs, longer and younger TEs are more likely to form dsRNA. This points to a relationship between the physical properties of TEs and their propensity to form structurally complex RNA molecules, such as dsRNA.

Finally, we compared the results of our two-step dsRNA purification with a method designed to map dsRNA interactions (PARIS). PARIS uses reversible psoralen crosslinking to identify RNA duplexes in living cells, including mouse ESCs (Lu et al. 2016). After high-throughput sequencing of RNA duplexes, chimeric reads are used to identify the specific regions of interacting RNA, at a single-nucleotide resolution. We reanalyzed this available data set focusing on transposable elements. Although most chimeras formed within rRNAs and mRNAs, we also observed 116,000 chimeras that contained TE sequences. However, only 13% of these chimeras were exclusively between TEs (TE-TE chimeras). Interestingly, TE-TE chimeras were solely detected for LINE and LTR classes, but not for DNA or SINEs (Fig. 4E). Evolutionary young LINE-1 subfamilies, including L1MdTf_II, L1MdTf_III, L1MdF_IV, and L1MdA_III, were the most frequently recovered in TE-TE chimeras. Young LTR elements, such as the rodent-specific IAPEz and the mouse-specific MERVL, were also among the top contributors (Fig. 4F). These data independently confirm our results that younger TE subfamilies, particularly those belonging to LINE and LTR classes, are more likely to form dsRNA. Next, we performed a direct comparison between our method and PARIS to test whether TE subfamilies that form more chimeras were also more enriched by dsRNA-IP. To this end, we annotated each TE subfamily with its PARIS TE-TE chimera count and examined the relationship with log_2_FC across the dsRNA IPs. In the dsRNA versus input comparison, we observed an inverse correlation; chimera-rich subfamilies showed lower fold enrichment, suggesting that dsRNA IP (J2) on its own is not enough to enrich for dsRNA. This relationship, however, reversed after RNase I treatment. Here, chimera-rich elements positively correlated with enrichment, indicating that RNase I treatment helps enriching for the subfamilies most engaged in dsRNA formation (Fig. 4G,H). These results suggest that RNase I treatment improves the isolation of transcripts that can form dsRNA interactions in cells. Lastly, we compared the evolutionary age. Again, we found that chimera-rich LINE subfamilies were among the most significantly enriched after RNase I treatment. This analysis independently confirms that younger TE subfamilies, particularly from LINE and LTR classes, are more likely to form dsRNA in mESCs.

## DISCUSSION

It is becoming increasingly evident that mammalian cells, much like viruses, can produce dsRNA and have evolved multiple mechanisms to prevent its accumulation or promote its disruption and degradation. Dysregulation of dsRNA metabolism is now recognized as a contributing factor to human disease, including autoimmune disorders and cancer (de Reuver and Maelfait 2024). Despite these advances, the identification of dsRNA remains challenging due to the lack of methods that specifically enrich for dsRNA. Current techniques often require a relatively high abundance of dsRNA for successful detection, favoring settings with high levels of dsRNA such as infections. The development of methods with an increased sensitivity for dsRNA detection and identification are important in the context of innate immunity as only a small number of dsRNA molecules in a few cells may be required to initiate a cascade of immune reactions.

Intriguingly, ESCs accumulate detectable amounts of endogenous dsRNA without triggering an antiviral response. Consequently, ESCs have been chosen as a model cell system to refine existing methods for dsRNA identification. Previous methods for cellular dsRNA identification have relied on dsRNA binding proteins or dsRNA-based antibody purification. Here, we demonstrate that using a two-step purification starting with dsRNA immunoprecipitation followed by RNase I treatment enriches for transposable elements. Although the addition of RNase I enhances dsRNA identification, complete depletion of ssRNA is not expected, as RNase activity may be limited by steric hindrance from the antibody or RNA-binding proteins present in the immunoprecipitate. Identified TEs exhibited higher levels of A-to-I editing, increased overlapping sense/antisense reads and are derived from longer and evolutionary younger elements. Overall, these data suggest that our two-step purification enriches for RNAs with features consistent with increased duplex formation and that they conceivably represent bona fide dsRNA.

Previous applications of dsRNA-based methodologies have identified both the mitochondria and the nucleus as potential sources of dsRNA in mammalian cells (Sadeq et al. 2021; Chen and Hur 2022; Cottrell et al. 2024). However, we propose that the origin of dsRNA varies depending on the cell type and the nature of the perturbation applied. We do not anticipate that dsRNA identities will be universally conserved across cell types, even when they originate from the same genome. For instance, PKR has been shown to interact with mitochondrial RNA, and inactivation of the mitochondrial exonuclease Pnpt1 leads to an accumulation of mitochondrial dsRNA in HeLa cells (Dhir et al. 2018; Kim et al. 2018). Nevertheless, dsRNA is generally generated by nuclear transcription and is enriched in TEs (Gázquez-Gutiérrez et al. 2021). In agreement, dsRNA-IPs from mouse ESCs showed a clear enrichment for TEs but not for mitochondrial RNAs.

One of the best recognized classes of TEs involved in dsRNA formation are SINEs. These elements are the most abundant TEs in mammalian genomes, with ∼1.4 and 1.5 million copies in the mouse and human genome, respectively. The successful expansion of SINEs is likely because they are short, noncoding elements, and therefore, generally well-tolerated even when inserted within genes. Also, SINE insertions are usually found in the noncoding regions of genes, including introns and 3′UTRs (Lander et al. 2001; Maquat 2020). Their density is so high that a typical human gene contains more than one Alu element, which may be inserted in sense and antisense orientation (inverted-repeat, IR-Alus), leading to dsRNA formation. The high abundance of SINEs copies, as well as high number of SINE-overlapping transcripts that are present in cells has made them a prime candidate for dsRNA formation across multiple cell types (Kim et al. 2014; Ahmad et al. 2018; Chung et al. 2018; Heinrich et al. 2019; Mehdipour et al. 2020). Although SINEs form dsRNA, they are unlikely to trigger an immune response because they are the main targets of mechanisms known to disrupt dsRNA structures, including ADAR1-mediated editing and DHX9 helicase activity (Aktaş et al. 2017; Levanon et al. 2024). Also, IR-Alus contain ∼20% mismatches within the duplex region (Ahmad et al. 2018). We hypothesize that these features render them more susceptible to RNase I treatment than sense/antisense LTR or LINE elements.

Our data suggest that in addition to SINEs, LTRs and LINEs are also major contributors to dsRNA formation in ESCs. We hypothesize that this is likely a direct consequence of the transcriptomic profile of ESCs. During the early stages of development, the expression of other types of full-length elements is temporarily allowed, while the epigenetic marks that silence TEs are remodeled. In ESCs, we observed that LINEs and LTRs are proportionally more resistant to RNase I treatment than SINEs. This is consistent with findings in other settings, where both LINE-1 and LTRs have been involved in dsRNA formation after TE transcription is derepressed, either by inactivating the HUSH complex or by triggering DNA demethylation (Chiappinelli et al. 2015; Roulois et al. 2015; Tunbak et al. 2020). Therefore, we conclude that under conditions where LINEs and LTRs can be expressed, these may also contribute to dsRNA formation. Also, we hypothesize that LINEs and LTRs may form dsRNA more efficiently than SINEs, as they tend to be longer, and thus the number of potential bases embedded in dsRNA structures is larger. The high levels of TE-derived dsRNA may explain the need to downregulate the innate immune responses, as these molecules are immunogenic ligands nucleic acid sensors including MDA5, PKR and TLRs (Gázquez-Gutiérrez et al. 2021; Witteveldt et al. 2025).

Interestingly, we observed A-to-I editing across all classes of transposons, including LINEs, LTRs, SINEs and to a lower extent, DNA. However, SINEs and in particular Alu elements, have been previously identified as the main target for ADAR1-mediated editing (Blow et al. 2004; Bazak et al. 2014; Herrmann et al. 2026). We hypothesize that the diversity in editing targets may be linked to the transcriptomic profile of cells. In somatic cells and adult tissues, full-length LINE or LTRs are usually not expressed, and cannot serve as ADAR1 substrates. Conversely, in ESCs, both LINEs and LTRs retrotransposons are expressed and form dsRNA and can therefore be edited at levels comparable to those observed in SINEs. The impact of this editing on dsRNA structure may differ among the various TE classes. IR-Alus generate shorter dsRNA stems (<300 bp) and contain more unpaired regions that are sensitive to RNase I treatment. In contrast, our findings indicate that LINE elements can potentially form dsRNA with an average length of 500 bp and extending up to 1 kb. Given the strong enrichment of both sense and antisense strands in LINEs, we predict that the dsRNA formed by these elements can be up to 1 kb long. Such long dsRNA segments are likely to form more stable duplexes which are less affected by editing, while shorter or less stable dsRNA structures, like those formed by SINEs, may be more susceptible to destabilization at similar levels of editing. Interestingly, the observed levels of editing are high, yet insufficient to disrupt the dsRNA structure enough to prevent recognition by the dsRNA antibody. Although this may not be relevant in the context of ESCs, the extent of editing could still be sufficient to evade cellular immune sensing.

In addition to length, the evolutionary age of TEs appears to be crucial for the formation of dsRNA. Somatic adult cells rarely express full-length young TEs because they retain the ability to retrotranspose. However, a different scenario unfolds during development. For instance, LINE and LTR elements, such as LINE- 1 and MusD, are still capable of transposition within the mouse genome (Ribet et al. 2004; Richardson et al. 2015) and about 3000 copies from the L1 subfamilies A, Tf and Gf remain active in mice (DeBerardinis and Kazazian 1999; Li et al. 2014). From the perspective of the selfish element, the optimal window for transposition is early development, before germline specification. In this manner, the new transposition event has the potential to be passed on to subsequent generations. Considering that younger elements have had less time to accumulate mutations and be eliminated through post-insertion selection, their transcription in both sense and antisense orientations is more likely to produce perfectly paired dsRNA.

The origin of antisense LINEs and LTRs transcripts remains uncertain. LINE-1 elements contain an antisense RNA-polymerase II promoter, which could potentially lead to dsRNA formation (Li et al. 2014). To our knowledge, no antisense promoters have been identified for LTRs or SINEs (Oomen et al. 2025). However, when mapping dsRNA-seq reads to the consensus sequences of these elements, antisense reads appear not to be confined to a specific region but are detected throughout the element. It is possible that the antisense RNA is co-transcribed from the promoter of its host gene. In humans, LINE-1 insertions within genes tend to be selected in the antisense orientation, possibly to mitigate the harmful effects of sense-oriented transcripts expressed as part of the host gene (Medstrand et al. 2002; Zhang et al. 2011; Flasch et al. 2019; Sultana et al. 2019). Similarly, selection appears to have favored antisense insertions for both LINE and LTR elements in mice, suggesting that expression of the antisense transcript could be driven by the promoter of a neighboring or the host gene (Zhang et al. 2011). If this model is correct, antisense transcripts containing LINE and LTR sequences would be constitutively co-expressed when embedded in other genes, with dsRNA formation occurring when sense copies are transcribed from their own promoter or as part of the host genes.

To conclude, we have developed a method to enrich and identify RNAs with features consistent with increased duplex formation and that these are likely to represent bona fide cellular dsRNA. This approach will be critical when revisiting previously described dsRNA species and confirming the identity of dsRNA in other contexts, such as autoimmune disorders or cancer.

## MATERIALS AND METHODS

### Cell lines and differentiation

The mouse embryonic stem cell (ESC) line v6.5 was obtained from Thermo Fisher (MES1402) and maintained in Dulbecco's Modified Eagle Medium (DMEM, Thermo Fisher 41966) supplemented with 15% heat-inactivated fetal calf serum (FCS, Thermo Fisher), 1× minimal essential amino acids (Thermo Fisher 11140035), 2 mM l-glutamine, 10^3^ U/mL leukemia inhibitory factor (STEMCELL Technologies 78056), and 50 µM 2-mercaptoethanol (Thermo Fisher 31350010). ESCs were cultured on 0.1% gelatin-coated plates and dissociated using 0.05% trypsin (Thermo Fisher 25200056). To differentiate, mouse ESCs were grown in ESC medium in the absence of LIF, and supplemented with 10 µM of retinoic acid (Sigma-Aldrich R2625). Cells received fresh medium daily for a minimum of 10 days to ensure successful differentiation.

### dsRNA transfections

Cells were transfected with the dsRNA analog poly(I:C) (InvivoGen, tlrl-pic) using Lipofectamine 2000 (Thermo Fisher 11668027). Transfections were performed in 6 and 24 well format for protein and RNA, respectively. At ∼80% confluency, cells were transfected using 0.5 µg/mL poly(I:C) and collected after 2 h for protein samples and 6 h for the RNA samples.

### RT-qPCR

Total RNA was extracted using TRI Reagent (MilliporeSigma), and cDNA was synthesized using M-MLV (Promega M1701) in accordance with the manufacturer's instructions. qPCR reactions were performed using GoTaq qPCR Master Mix (Promega A6001) using previously published primers (Supplemental File 1) on a QuantStudio 5 (Thermo Fisher). Data were analyzed using QuantStudio Design & Analysis software. Differences were analyzed by single factor ANOVA to calculate significant differences among comparisons, followed by a Tukey HSD. (*) *P* ≤ 0.05, (**) *P* ≤ 0.01, (***) *P* ≤ 0.001.

### Western blots

Cells were washed twice with PBS followed by lysis in RIPA buffer (50 mM Tris-HCl, pH 7.4, 1% Triton X-100, 0.5% Na-deoxycholate, 0.1% SDS, 150 mM NaCl) supplemented with protease (Sigma-Aldrich 11873580001) and phosphatase inhibitors (Sigma-Aldrich P7526). Samples were sonicated and centrifugated to shear nucleic acids and clarify the lysates. Protein concentrations were determined using the BCA assay (Thermo Fisher 23225) and equal amounts mixed with LDS sample buffer (Thermo Fisher NP0007) and Sample reducing agent (Thermo Fisher NP0009), and run on 4%–12% Bis-Tris precast gels (Thermo Fisher). Proteins were transferred to nitrocellulose membrane using wet transfer (Mini Blot module, Thermo Fisher) according to the manufacturer's recommendations. Membranes were blocked for 1 h at room temperature in TBS-T (0.1% Tween-20) and 5% fetal calf serum before overnight incubation at 4°C with primary antibody, followed by the appropriate secondary antibody for 1 h at RT. Antibodies used were anti-phospho PKR (Merck 07-886), anti-phospho IRF3 (Cell Signalling D6O1M), anti-phospho Eif2a (Cell Signalling D9G8), anti-PKR (Santa Cruz sc6282), anti-IRF3 (BioLegend 655701), anti-tubulin (Calbiochem CP06), anti-GAPDH (Merck CB1001), anti-mouse 680RD (LICORbio 925-68070), anti-mouse 800CW (LICORbio 926-32210), anti-rabbit 680RD (LICORbio 925-68071), anti-rabbit 800CW (LICORbio 926-32211). Membranes were dephosphorylated with 57 U/mL alkaline phosphatase (Promega M1821) in 50 mM Tris-HCl (pH 7.5), 2 mM MgCl_2_ and 0.1 mM DTT overnight at 4°C. Protein bands were visualized on a Li-cor imaging system (LICORbio) and quantified using the Li-cor software. Expression levels were calculated normalized to tubulin or GAPDH.

### Flow cytometry

Cells were dissociated using 0.05% trypsin, washed in PBS and resuspended in FACS buffer (PBS with 1% FBS). Cells were pelleted and resuspended in Fixation buffer (BioLegend 420801) and incubated for 20 min at 4°C, washed twice with Intracellular Staining Permeabilization Wash Buffer (BioLegend 421002). Cells were washed once with RNase III reaction buffer (NEB, ShortCut) in the absence of MnCl_2_ and incubated for 45 min at 37°C in 100 µL complete reaction buffer with (2 µL) or without RNase III. Cells were washed twice with Intracellular Staining Permeabilization Wash Buffer and incubated overnight with the anti-dsRNA antibody J2 (English & Scientific Consulting) in Intracellular Staining Permeabilization Wash Buffer (1:400). After incubation, cells were washed twice with Intracellular Staining Permeabilization Wash Buffer and incubated with anti-mouse Alexa Fluor 647 (BioLegend 405322) for 1 h at room temperature and washed three times with Intracellular Staining Permeabilization Wash Buffer before finally resuspending the cells in FACS buffer. Cells were analyzed using a MACS Quant Analyzer 10 (Miltenyi), and data were processed using FlowJo software (TreeStar).

### Immunofluorescence

Glass coverslips were coated with 0.03 μg/mL laminin (Sigma Aldrich L2020) for 30 min at room temperature, aspirated and washed with PBS before addition of cells. For immunofluorescence, cells were washed once with PBS and fixed with 4% paraformaldehyde (Sigma Aldrich) for 10 min at RT. Cells were washed thrice with PBS, and the cell membrane was stained with 2 μg/mL CF488A-conjugated lectin antibody (Biotinum 29022) for 30 min at RT. Cells were washed and permeabilized with 0.2% Triton-X100 (Sigma Aldrich) and incubated in blocking buffer (5% FCS, 0.1% Triton-X100). Cells were incubated overnight at 4°C with J2 antibody (1:300) diluted in blocking buffer. Cells were washed thrice with blocking buffer before incubation with anti-mouse Alexa Fluor 647 diluted in blocking buffer (1:4000) and incubated for 45 min at RT. DAPI (Sigma Aldrich) was diluted to 10 μg/mL to stain DNA for 5 min at RT. Cells were again washed thrice with blocking buffer before a final wash with PBS prior to mounting the coverslips onto microscope slides with ProLong Diamond reagent (Invitrogen P36965). Image acquisition was performed using a Zeiss Axio Imager Z2. FIJI (https://www.nature.com/articles/nmeth.2019) was used for image analysis.

### RNA-seq of ESCs and differentiated cells

For sequencing, cells were differentiated as described above and appropriate numbers of ESCs and differentiated cells were collected and lysed in DNA/RNA shield (Zymo). Extracted RNA was purified by poly(A) mRNA capture and converted to cDNA using poly(dT)VN primers, followed by tagmentation, library indexing and amplification. Libraries were sequenced using an Illumina NovaSeq X Plus instrument. Differential gene expression was calculated using 3′ end counting and pipeline described here (https://plasmidsaurus.com/technical-documentation/rna). In addition, the previously published RNA high-throughput data set of mESCs differentiation (https://www.ebi.ac.uk/biostudies/arrayexpress/studies/E-MTAB-4904) was used (Sladitschek and Neveu 2019).

### DsRNA immunoprecipitation and sequencing

ESCs were detached with 0.05% trypsin and washed twice with PBS. For each IP, one 15 cm plate was used. Cells were lysed in IP buffer (50 mM Tris pH 7.5, 150 mM NaCl, 1 mM EDTA, 1% Triton X-100), supplemented with protease inhibitors (Roche 11836170001), 80U RNase inhibitor (Promega N2518), 10U DNase (Promega RQ1) on ice for 30 min, followed by centrifugation at 17,000*g* for 15 min at 4°C to clarify the samples. Protein G magnetic beads (50 µL, Invitrogen 10004D) were washed thrice with IP buffer, and mixed with 5 µg of either anti-dsRNA J2 antibody or IgG2a-κ antibody as a negative control. The conjugated beads were washed thrice with cold IP buffer, and incubated with cell lysates for 4 h at 4°C. Beads were washed three times with cold IP buffer and incubated with either 100U RNase I (NEB M0243S) or 2U RNase III (NEB M0245S), in 100 µL of the manufacturer's supplied buffers for 20′ at 37°C. Mock-treated samples were resuspended in RNase III buffer without RNase III. After washing three times with IP buffer, RNA was isolated using TRIzol LS reagent (Sigma Aldrich T3934) and 0.3 M sodium acetate, according to the manufacturer's instructions.

### DsRNA computational analysis

Total RNA quality was assessed on the Agilent 2100 Electrophoresis Bioanalyzer Instrument (Agilent G2939AA). RNA was quantified using the Qubit 2.0 Fluorometer (Thermo Fisher Q32866). DNA contamination was quantified using the Qubit dsDNA HS Assay Kit and confirmed to be <6% (Thermo Fisher Q32854). Libraries for total RNA sequencing were prepared using the NEBNext Ultra 2 Directional RNA Library Prep Kit (Illumina E7760) and the NEBNext rRNA Depletion Kit (Human/Mouse/Rat) (Illumina E6310), according to the provided protocol. Sequencing was performed on the NextSeq 2000 platform (Illumina 20038897) using NextSeq 2000 P3 Reagents (200 cycles, 2 × 100 bp) (20040559). Paired-end RNA-seq reads were adapter-trimmed using Cutadapt (v5.1) with a minimum length of 50 bp and NextSeq-specific quality trimming (Martin 2011). Reads were aligned to the mouse reference genome (mm39) using STAR (v2.7.11b) with end-to-end alignment, retaining up to 1000 alignments per multimapping read (‐‐outFilterMultimapNmax 1000, ‐‐outSAMmultNmax 1000) and a maximum of three mismatches (‐‐outFilterMismatchNmax 3) (Dobin et al. 2013). Sorted BAM files were generated and indexed using SAMtools (v1.23.1) (Dobin et al. 2013). Strand-specific coverage tracks were produced with deepTools (v3.5.1) (bamCoverage) using CPM normalization and an effective genome size of 2.6 Gb (Dobin et al. 2013). Sense and antisense signals were intersected using BEDTools (v2.31.0) to quantify strand overlap (Quinlan and Hall 2010). dsRNA levels were quantified using an overlap metric, defined as the proportion of antisense signal overlapping sense transcription (overlap/sense signal). Overlap was calculated as the minimum coverage between strands at each position, weighted by genomic span. Metrics were computed genome-wide and within TE classes using merged RepeatMasker-derived annotations.

Reads were assigned to genomic features using featureCounts (v2.1.1) with multimapping enabled (Liao et al. 2014). Gene-level counts were summarized by transcript biotype, while repeat counts were grouped by TE family and class using custom annotations. Counts were normalized per sample to relative proportions.

Transposable element (TE) and gene counts were quantified using TEcount (TEtranscripts v2.2.3) in multimapping mode with strandedness set to reverse, using gene and TE annotations (Jin et al. 2015). In this mode, TEcount applies an expectation-maximization algorithm that fractionally distributes multimapping reads across individual TE copies, then sums counts to the subfamily level. All TE differential expression analyses are performed at the TE subfamily level. Differential expression analysis was performed in R (v4.5.0) using DESeq2 (v1.44.0) (Love et al. 2014). Size factors were estimated from gene counts only and applied to TE counts to avoid bias from repetitive elements. Lowly expressed features (mean count <10) were excluded. Differential expression was assessed using a negative binomial model, and significance was defined as |log_2_FC| > 1 and adjusted *P* < 0.05. Variance-stabilizing transformation (VST) was applied for downstream visualization.

For RNA editing analysis, trimmed RNA-seq reads were aligned to the reference genome using STAR (Dobin et al. 2013), retaining only uniquely mapped reads (‐‐outFilterMultimapNmax 1) and allowing up to 10 mismatches per read, with a maximum mismatch rate of 5% of read length. Base counts at all covered A and T positions within RepeatMasker-annotated transposable elements were obtained using samtools mpileup (v1.23.1), requiring a minimum base quality of 30. Positions where a single nonreference nucleotide accounted for >90% of reads were excluded as likely genomic variants. A-to-I editing was quantified using a coverage-weighted editing index following the framework described by Roth et al. (2019), in which editing levels are calculated as the ratio of summed edited reads to summed informative reads across all qualifying positions within a genomic region. Editing indices were calculated separately for LINE, LTR, SINE, and DNA classes, as well as for all retrotransposons combined. Corresponding indices for nonediting substitutions (A-to-C, A-to-T, T-to-A, and T-to-G) were computed as specificity controls.

### TE-TE chimera analysis

RNA proximity-ligation data by psoralen crosslinking (PARIS) in mESCs was obtained from SRA accessions SRR2814766, SRR2814767, SRR2814768; three replicates (Lu et al. 2016). Reads were mapped and chimeras identified with hyb2 (https://github.com/Jylau14/hyb2), a bowtie2-based reimplementation of the hyb pipeline (Travis et al. 2014), using a custom mouse reference combining Repbase/DFAM transposable-element/repeat consensus sequences, mouse rRNA, and RefSeq “select” mRNAs (database v4). To handle the highly multimapping repeat-derived reads, three nondefault settings were used: bowtie2 reported up to *k* = 100 alignments per read, and up to *h* = 1000 hits per read were retained for chimera calling, and sense and antisense-mapped reads were allowed for chimera calling (other parameters default: *e*-value ≤0.1, maximum bit overlap 4 nt). Single-read and chimeric-read composition, by RNA class and TE subclass (from the RepeatMasker name#class/family annotation), were tabulated from the SAM and deduplicated .hyb files.

Hyb2 output files were further processed in R. Analyses were restricted to TE-TE chimeras (both interacting arms annotated as transposable elements), which were summarized by TE class and subfamily. Chimeric read counts for each TE subfamily were then integrated with J2 dsRNA-seq differential expression results.

## DATA DEPOSITION

All sequencing data sets have been deposited under the SRA BioProject PRJNA1441232. Raw data can be accessed at https://www.ncbi.nlm.nih.gov/bioproject/?term=PRJNA1441232.

## SUPPLEMENTAL MATERIAL

Supplemental material is available for this article.
